# Supplementary material for: Occurrence, Biological Characteristics, and Annual Dynamics of Atherigona orientalis (Schiner 1968) (Diptera: Muscidae) in China
Source: Insects. 2025 Sep 4;16(9):931. doi: 10.3390/insects16090931 (PMC12470737; doi:10.3390/insects16090931)
Supplement: Supplementary file 1 [file insects-16-00931-s001.zip › insects-3775218-supplementary.pdf]

Occurrence, Biological characteristics, and Annual dynamics of *Atherigona orientalis* (Schiner 1968) (Diptera: Muscidae) in China

Zihao Zhou, Yujie Luo, Jiawei Qin, Xintong Wang, Shuaijun Ning, Jing He, Qiong Zhou *

College of Life Sciences, Hunan Normal University, Changsha, China, 410006

* Corresponding author.

E-mail addresses: zhoujoan@hunnu.edu.cn (Q. Zhou)

Supplementary Tables

Table S1 Information on fruit and vegetable sampling in different regions of Hunan Province

Cities and Autonomic Prefecture	District or county	Longitude and latitude	Collection date	Host plant
Changde	Lixian	111°74′ N<br>29°6′ E	2020.9 and 2022.9	Chili pepper, bitter melon, persimmon
Changsha	Changshaxian	113°36′ N<br>28°12′ E	2022.9	Chili pepper, bitter melon, luffa
	Kaifu	112°99′ N<br>28°21′ E	2022.8	Chili pepper
	Ningxiang	112°33′ N<br>28°08′ E	2020.8<br>2020.10<br>2022.9	Chili pepper, eggplant

	Liuyang	113°63′ N<br>28°16′ E	2020.9	Luffa, bitter melon
	Yuelu	112°95′ N<br>28°2′ E	2022.8	Chili pepper
	Yuhua	113°03′ N<br>28°18′ E	2022.9	Chili pepper
Chenzhou	Zixing	113°23′ N<br>25°97′ E	2021.10	Mandarin orange
Huaihua	Huitong	109°75′ N<br>26°96′ E	2020.11<br>2022.7	Chili pepper, melon, bitter melon
	Xupu	110°63′ N<br>27°56′ E	2020.10<br>2022.8	Chili pepper, eggplant, bitter melon, luffa, fig
	Yuanling	110°45′ N<br>28°78′ E	2022.9	Chili pepper, bitter melon
	Zhijiang	109°77′ N<br>27°46′ E	2020.8<br>2021.9<br>2022.7	Chili pepper, bitter melon, tomato, melon, pumpkin, winter melon, eggplant
	Chenxi	110°18′ N<br>28°02′ E	2020.11<br>2021.9	Bitter melon
	Zhongfang	109°93′ N<br>27°40′ E	2020.10	Luffa
Hengyang	Hengdong	112°97′ N<br>27°29′ E	2020.11<br>2022.8	Chili pepper, eggplant, bitter melon
	Qidong	111°52′ N<br>26°53′ E	2021.9	Yellow peach
Loudi	Shuangfeng	112°15′ N	2021.9	Chili pepper, bitter melon, luffa, yellow peach, plum

		27°44′ E	2022.7	
	Lianyuan	111°86′ N<br>27°85′ E	2022.7	Chili pepper, bitter melon, luffa
	Louxing	112°09′ N<br>27°78′ E	2022.8	Eggplant, bitter melon, luffa, cucumber
Shaoyang	Chengbu	110°32′ N<br>26°04′ E	2020.10<br>2022.8	Bitter melon, pear
	Daxiang	111°47′ N<br>27°25′ E	2020.10<br>2022.9	Chili pepper, eggplant, bitter melon, luffa, tomato
	Xinshao	111°22′ N<br>27°43′ E	2020.10<br>2021.9<br>2022.9	Chili pepper, eggplant, zucchini, luffa, cattail
	Shaodong	11°49′ N<br>27°20′ E	2022.9	Chili pepper, eggplant, luffa
	Longhui	111°04′ N<br>27°12′ E	2022.8	Chili pepper, eggplant, bitter melon
Xiangtan	Yuhu	112°77′ N<br>27°91′ E	2020.10<br>2021.11	Chili pepper, eggplant
	Xiangtanxian	112°95′ N<br>27°78′ E	2020.10	Chili pepper, eggplant, luffa, pumpkin
Xiangxi	Baojing	109°24′ E<br>28°36′ N	2020.11	Kiwi
	Fenghuang	109°43′ N<br>27°92′ E	2020.10、11	Kiwi
	Jishou	109°73′ N<br>28°29′ E	2022.9	Chili pepper, bitter melon, tomato, pumpkin

	Luxi	110°21′ N<br>28°22′ E	2020.11<br>2021.11	Chili pepper, bitter melon, luffa, pumpkin, winter melon
Yiyang	Heshan	112°40′ N<br>28°58′ E	2022.9	Bitter melon, yellow peach
	Taojiang	112°16′ N<br>28°52′ E	2022.7	Chili pepper, eggplant, bitter melon
Yueyang	Miluo	113°16′ N<br>28°78′ E	2022.9	Chili pepper
	Pingjiang	113°57′ E<br>28°43′ N	2020.11	Chili pepper, luffa, pumpkin, winter melon
	Xiangyin	112°30′ N<br>28°30′ E	2020.11	Chili pepper
Yongzhou	Daoxian	111°61′ N<br>25°54′ E	2022.7	Chili pepper, eggplant, melon
	Jiangyong	111°34′ N<br>25°27′ E	2021.8	Chili pepper, zucchini, bitter melon, tangerine
Zhangjiajie	Cili	110°98′ N<br>29°38′ E	2020.10<br>2022.9	Chili pepper, eggplant, bitter melon, luffa, pumpkin
	Yongding	111°47′ N<br>27°25′ E	2022.9	Chili pepper, eggplant, luffa, pumpkin, peach, red date
Zhuzhou	Liling	113°50′ N<br>27°67′ E	2020.10	Pumpkin
	Yanling	113°78′ N<br>26°50′ E	2022.9<br>2022.10	Chili pepper, yellow peach

Table S2 Specific information about the experimental sties

Site number	Area	Longitude and latitude	Number of yellow cardstocks
1	2 m × 8 m	112°957′ E, 28°194′ N	5
2	3 m × 5 m	112°957′ E, 28°195′ N	5
3	40 m × 20 m	112°955′ E, 28°198′ N	5
4	20 m × 20 m	112°955′ E, 28°197′ N	5
5	50 m × 15 m	112°955′ E, 28°196′ N	5
6	10 m × 8 m	112°951′ E, 28°189′ N	5
7	15 m × 2 m	112°951′ E, 28°189′ N	5

Table S3 The selection of different colored sticky plates by the *Atherigona orientalis*

Color	First month	Second month	Third month	Total
Red	9%	8%	10%	9%
Orange	9%	18%	15%	15%
Yellow	37%	22%	29%	29%
Green	34%	35%	32%	34%
Blue	0%	6%	5%	4%
Purple	6%	6%	5%	5%

.

Table S4 The population dynamics of the *Atherigona orientalis* in the surrounding areas of Changsha City

Data	Average female number	Average male number	Maximum temperature	Minimum temperature
2022.3.7	0.171	0	18	9
2022.3.14	0.200	0.029	25	15
2022.3.25	0.229	0.057	18	10
2022.4.11	0.171	0.143	24	13
2022.4.21	0.286	0.114	20	12
2022.4.30	0.229	0	26	18
2022.5.10	0.257	0.114	27	17
2022.5.20	0.314	0.143	24	17
2022.6.1	0.286	0.200	27	22
2022.6.10	0.257	0.257	30	23
2022.6.21	0.600	0.029	30	23
2022.6.30	0.543	0.143	34	26
2022.7.10	1.886	1.029	34	26
2022.7.20	2.629	2.686	37	27
2022.7.31	1.114	0.829	37	27
2022.8.10	2.143	2.229	39	29
2022.8.20	1.486	1.229	39	29
2022.8.31	0.686	0.629	37	28
2022.9.10	0.657	0.457	33	23
2022.9.20	0.343	0.257	35	23
2022.9.30	0.286	0.200	31	21
2022.10.10	0.029	0	24	17
2022.10.20	0.086	0.029	24	13
2022.10.31	0.057	0	26	16

2022.11.10	0.029	0.029	25	14
2022.11.20	0	0	19	13
2022.11.30	0	0	18	10
2023.2.20	0	0	15	5
2023.3.10	0.057	0.057	21	9
2023.3.20	0.029	0.029	17	9
